# Porcine circovirus 4 status of domestic pigs and wild animals in Poland

**DOI:** 10.2478/jvetres-2026-0044

**Published:** 2026-08-06

**Authors:** Agata Augustyniak, Ewelina Czyżewska-Dors, Dominik Łagowski, Iwona Giska, Małgorzata Pomorska-Mól

**Affiliations:** 1 Department of Preclinical Sciences and Infectious Diseases, Department of Internal Diseases and Diagnostics, Faculty of Veterinary Medicine and Animal Sciences, Poznań University of Life Sciences, 60-637 Poznań, Poland; 2 Department of Internal Diseases and Diagnostics, Faculty of Veterinary Medicine and Animal Sciences, Poznań University of Life Sciences, 60-637 Poznań, Poland; 3 Centre for Research on Ecology and Forestry Applications, 08193 Cerdanyola del Vallès, Spain

**Keywords:** porcine circovirus 4, pig, wild boar, wild ruminants, Poland

## Abstract

**Introduction:**

Given the threat that porcine circovirus (PCV) 2 and PCV3 pose to the pig industry, the epidemiology of emerging PCV4 warrants further investigation. Until 2023, PCV4 had only been confirmed in Asia, with the majority of reports originating from China. More recently, PCV4 DNA has also been detected in samples from Spain and the United States, indicating a potentially broader geographic distribution. Furthermore, PCV4 DNA has been identified in samples from other animal species. This study aimed to evaluate the presence of PCV4 in samples collected from domestic pigs, wild boars and wild ruminants in Poland using its *Rep* (replicase) gene.

**Material and Methods:**

A total of 474 serum samples from domestic pigs and 226 blood and internal organ samples (lymph nodes, spleen and lungs) from 20 wild boars, 33 fallow deer, 4 red deer and 6 roe deer were tested using qPCR to detect the presence of PCV4 DNA.

**Results:**

No PCV4 was detected in any of the samples.

**Conclusion:**

The presence of PCV4 in other populations or regions of Poland cannot be excluded. Further research is needed to monitor the epidemiological situation in the country, particularly in light of emerging reports confirming the presence of the virus in Europe.

## Introduction

To date, four porcine circoviruses (PCV) have been identified. While PCV1 is non-pathogenic, PCV2 infections in pigs cause several clinical manifestations and have a significant economic impact (18, 22). Porcine circovirus 3 is considered pathogenic in pigs, with two major outcomes of PCV3 infections believed to be reproductive and systemic diseases (17). The pathogenicity and significance of PCV4, a virus first identified in 2019 (31), remain poorly understood. Although the DNA of this new virus has been found in samples from pigs showing clinical symptoms of respiratory, digestive, reproductive, nervous and systemic disorders, strong evidence of its pathogenic role is lacking (10, 12, 31). Based on experiences with PCV2 and PCV3, the potential role of PCV4 as a disease agent in pigs cannot be ruled out. The epidemiology of these emerging pathogens, which pose a potential threat to porcine health, requires further investigation.

Initially, PCV4 differs in distribution compared to PCV2 and PCV3, which are spread worldwide. Until 2023, PCV4 had been confirmed only in Asia, with the majority of reports originating from China, where it had been widely prevalent for some time, particularly in certain provinces (12). However, in 2023, the presence of PCV4 DNA was confirmed in pigs and wild boars in Spain, indicating that this virus has a broader distribution (9). This was subsequently confirmed by another study, which reported the detection of PCV4 DNA in various pig tissues in the United States (14). In addition, retrospective studies have tracked the circulation of PCV4 in China, confirming the presence of anti-PCV4 antibodies and viral DNA in samples collected in 2008 and 2012, respectively (7, 12). Available results indicate that PCV4 had also been circulating in Spain before 2019 (9, 10). For this country, the slow but progressive spread of PCV4 has recently been proven (10). Although it is impossible to draw firm conclusions because of the limited data available, Holgado-Martín *et al*. (10) suggest that Spain could also play a more significant role in the virus’ spread. The uncertainty regarding the worldwide spread and epidemiology of PCV4 necessitates additional studies assessing its circulation (10).

Like PCV1, 2 and 3 infections (22), PCV4 infections have also been reported in animals other than pigs (26, 27, 28, 29, 32). For instance, PCV4 DNA has been detected in samples obtained from wild boars (9, 10, 14, 27). This should not come as a surprise, as domestic pigs and wild boars are classified as the same species, *Sus scrofa*, and many pathogens that infect pigs also circulate in wild boars. Holgado-Martín *et al*. (11) considered wild boars a potential reservoir of PCV4 (11), and recently published a study indicating frequent PCV4 strain exchange between wild boars and pigs kept outdoors (10). Additionally, wild boars are reservoirs for various pathogens that can be transmitted to other animals. Wild boars share their habitats with different animal species, in Poland including fallow deer (*Dama dama*), roe deer (*Capreolus capreolus*) and red deer (*Cervus elaphus*). As shown elsewhere, these species can become infected with other PCVs (2, 3); therefore, it is reasonable to test whether PCV4 also circulates in these wild ruminants.

This study aimed to assess the presence of PCV4 in samples obtained in Poland from domestic pigs and four species of wild animals: wild boar, fallow deer, roe deer and red deer with the use of a qPCR targeting its *Rep* (replicase) gene.

## Material and Methods

### Samples

The present study included 474 serum samples collected from pigs between 2023 and 2024 and archived in the Laboratory of the Department of Preclinical Sciences and Infectious Diseases in the Faculty of Veterinary Medicine and Animal Sciences of Poznań University of Life Sciences. The samples originated from 10 commercial farrow-to-wean and farrow-to-finish farms located in six provinces (voivodeships) (Fig. 1). The farms differed in size (40–5,400 sows), herd health and hygiene status; however, all pigs were kept indoors, and all biosecurity measures required by law were implemented. Blood was collected from the *vena cava cranialis* or *vena jugularis* into clot activator tubes by the herds’ veterinarians for routine monitoring of the pigs’ health status. In total, 168 samples from piglets, 100 from weaners, 100 from fatteners and 106 from sows were randomly selected and included in the study. To obtain serum samples, blood was centrifuged at 2,500 × *g* for 15 min at 4°C, and the collected sera were stored at –70°C until analysis.

**Fig. 1.**
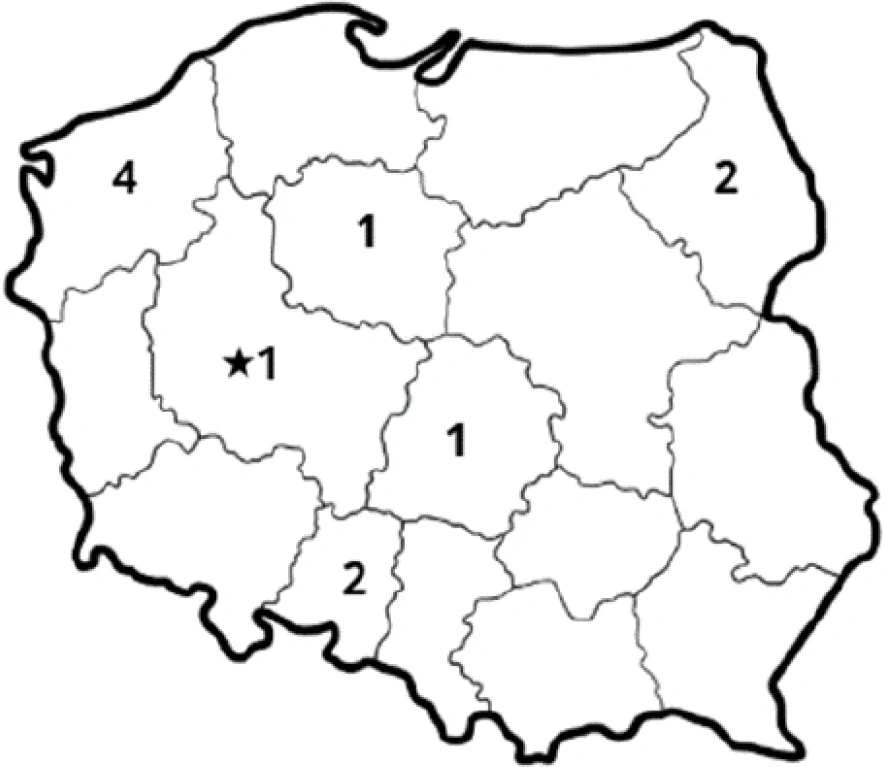
Number of pig farms sampled from each voivodeship for porcine circovirus 4 detection. The star indicates the voivodeship from which wildlife samples were obtained

In addition, 226 samples from four species of wild animals (wild boar, fallow deer, roe deer and red deer) culled in the Wielkopolskie voivodeship (starred in Fig. 1) were included in this study. Samples were collected *post mortem* by certified hunters. The hunting of animals complied with the Polish hunting law on animal protection. Samples from 20 wild boars, 33 fallow deer, 6 roe deer and 4 red deer were subjected to laboratory analyses. Samples of blood, lymph nodes, spleen and lungs were collected from each animal, if available. The exact number of samples investigated for each species is presented in Table 1. Blood samples were collected as blood clots from a main vein of the animal’s trunk or chest cavity. The remaining samples were harvested during evisceration of the carcass. The samples were transported to the laboratory under refrigeration. The blood and internal organs were stored at –70°C, collectively with the remaining samples, until analysis.

**Table 1.** Characteristics of samples collected from wild animals and analysed for porcine circovirus

Species	Serum	Lymph nodes	Spleen	Lung	Total
Wild boar (*Sus scrofa*)	18	16	20	20	74
Fallow deer (*Dama dama*)	30	19	32	33	114
Roe deer (*Capreolus capreolus*)	6	4	6	6	22
Red deer (*Cervus elaphus*)	4	4	4	4	16

### Laboratory analyses

Viral DNA was extracted from serum, blood and tissue homogenates using the Viral DNA/RNA Kit (A&A Biotechnology, Gdańsk, Poland) according to the manufacturer’s instructions. Each tissue was suspended in PBS at 20% and mechanically homogenised. The obtained homogenate was subsequently centrifuged (3,000 × *g* for 10 min at 4°C), and 150 μL of each supernatant was subjected to genetic material extraction. Extracted DNA was stored at –70°C until analysis.

The extracted DNA was used as a template for a qPCR targeting the PCV4 *Rep* gene. The primers and probe were newly designed based on PCV4 sequences available at the NCBI to amplify a 106-base-pair (bp) fragment of the *Rep* gene, and to account for polymorphisms in the reported *Rep* sequences. Primers were designed using Primer3Plus (23) (Table 2). Owing to the lack of available virus isolate, a 130-bp *Rep*-gene fragment of the GenBank reference sequence NC_055580.1, PCV4 open reading frame 1, was chemically synthesised (Institute of Biochemistry and Biophysics, Polish Academy of Science, Warsaw, Poland), cloned in pCRScript Amp SK plasmid (Promega, Madison, WI, USA) and used as a positive control.

**Table 2.** Primers and probes designed for the study

Name	Type	Sequence	Length (bp)	Position
PCV4 F	Forward Primer	GAAAGCGCAGCGACCTTA	18	397–414
PCV4 R	Reverse Primer	CCACGCCCATACCTTATAAAT	21	482–502
PCV4 probe	Probe	6-Fam-TGGCCCGTCGTGAGTTCCCGTCT-BHQ-1	20	459–479
PCV4 control	Positive control	GAAAGCGCAGCGACCTTAAAGCGGCTGTGGCCGCCCTGAATGCCGG CAGCTCAATGAGTGAAGTGGCCCGTGAGTTCCCGTCTGTATTTATAA GGTATGGGCGTGGCCTCCGGGACTACGTCATTACGC	130	397–526

The reaction mix included 7.3 μL of the extracted DNA, 15 μL of IndiMix JOE (Indical Bioscience, Leipzig, Germany), 1 μL of each primer (20 μM), 0.5 μL of probe (10 μM) and 0.2 μL of internal control template. The thermal profile of the real-time PCR reaction was as follows: 95°C for 2 min followed by 40 cycles of 95°C for 5 s and 60°C for 30 s. The reactions were performed in a CFX Opus thermocycler (Bio-Rad Laboratories, Hercules, CA, USA). The specificity was assessed by testing several samples positive for important swine pathogens, including PCV1–4. Amplification of serial dilutions of the positive control revealed a reaction efficiency of 98% and a limit of detection of 5,000 copies per reaction.

## Results

In total, 226 samples from four wildlife species, including wild boar and three deer species, comprising blood, lymph nodes, spleen and lung tissues, as well as 474 serum samples from domestic pigs, were analysed. None of the evaluated samples tested positive for PCV4 DNA. Successful amplification of PCV4 positive control DNA (Fig. 2) and internal control (Fig. 3) indicated the absence of significant PCR inhibition. The findings of the present study indicate no evidence of PCV4 circulation in the examined animal populations during the study period.

**Fig. 2.**
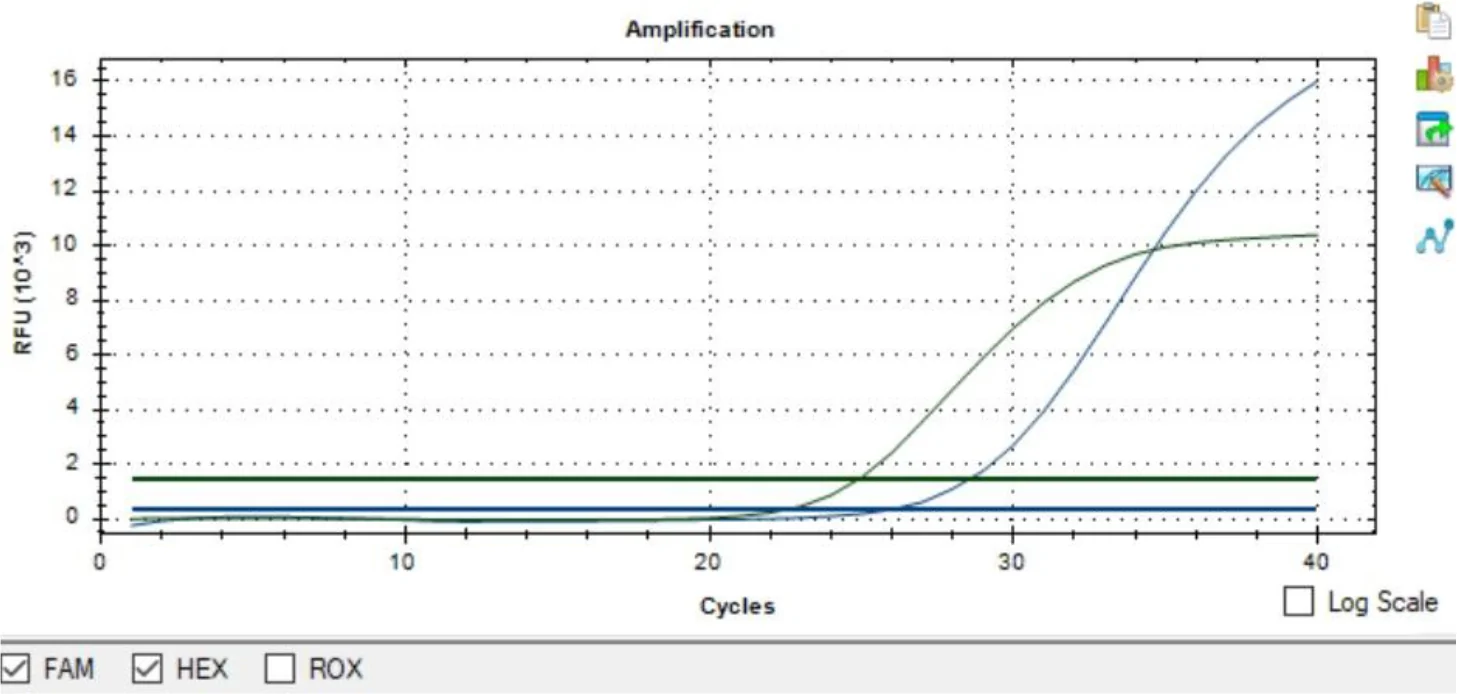
Representative real-time PCR amplification plot for PCV4 detection. The blue curve represents amplification of the PCV4 positive control detected in the FAM channel, while the green curve represents amplification of the internal control detected in the HEX channel

**Fig. 3.**
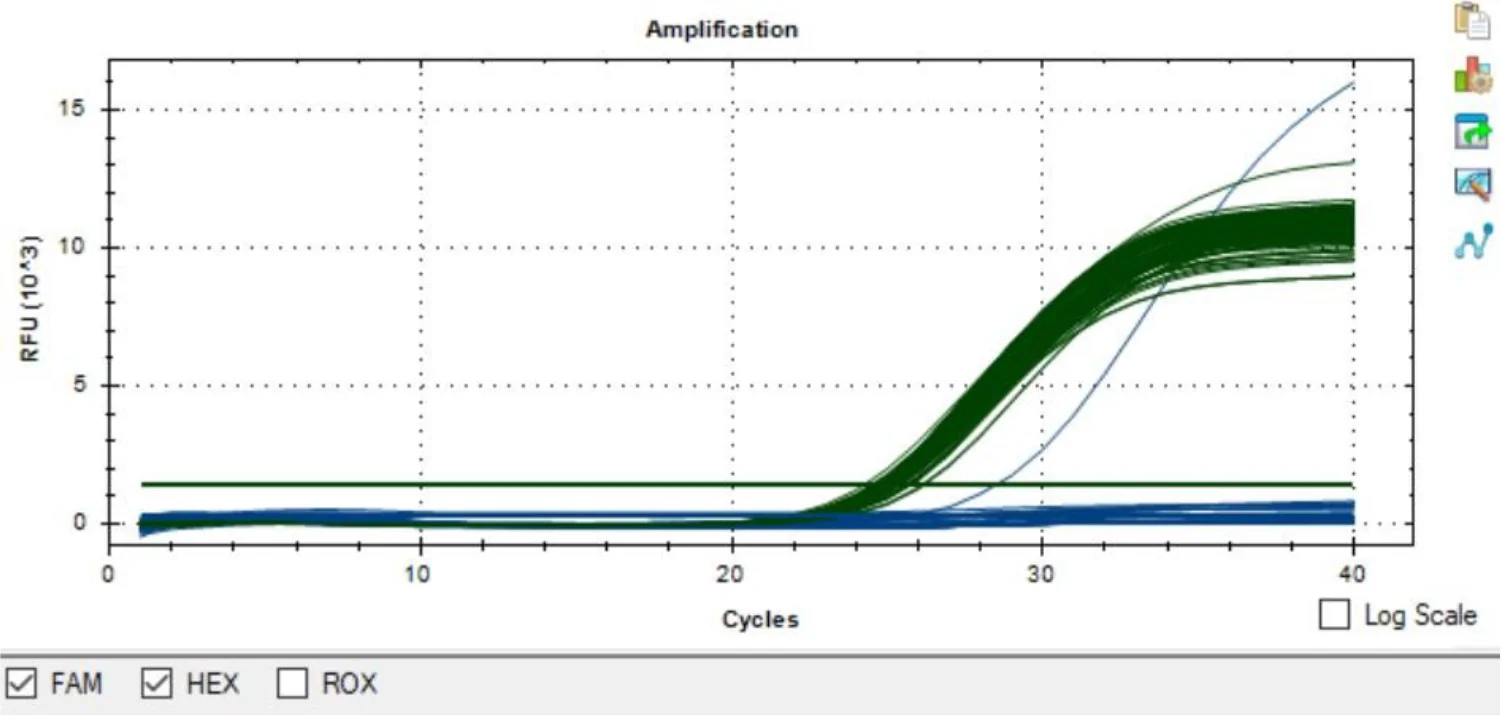
Representative real-time PCR amplification plot for PCV4 detection. The blue curve represents amplification of the PCV4 positive control in the FAM channel, while the green curves represent amplification of the internal control (IC) in the HEX channel in PCV4-negative samples. Successful IC amplification confirmed the validity of the negative results and indicated the absence of significant PCR inhibition

## Discussion

None of the evaluated samples tested positive for PCV4 DNA. Studies assessing and generally finding the circulation of PCV4 have been conducted in several countries so far, including China (7, 8, 12, 21, 27, 30, 31), South Korea (13, 15), Thailand (where it was not found in one instance (1) and found in another (19)), Malaysia (20), Spain (9, 10, 11), Italy (where it was not found (5)), the United States (14), Colombia (another investigation which failed to detect it (24)) and Poland (likewise without detection (4)). As already mentioned in the introduction, the highest number of published studies regarding PCV4 genetic material detection in pigs originate from China, where researchers reported PCV4-positive detection rates ranging from 1.69% to 45.39% (8, 12) and where viral DNA was detected in several provinces (25). The authors of one study, however, suggest that because of the large-scale movement of live pigs across China over the past decade, PCV4 is likely present in the majority of the country’s provinces (12). This assumption is supported by results from a seroprevalence study, in which sera from 1,790 pigs raised in 18 Chinese provinces were tested by ELISA for anti-PCV4 antibodies (7). Positive samples were detected in all provinces except one, with seroprevalence ranging from 9.72% to 83.33% across provinces, for an overall seroprevalence of 43.97% (7). In the remaining Asian countries where PCV4 has been detected in pigs, observed positivity rates were lower. Two studies have confirmed the circulation of PCV4 in pigs in South Korea (13, 15). In the first one, 3.28% of the analysed samples tested positive, and while the positivity rate was low, PCV4 DNA was detected in 6 out of 9 surveyed provinces (15). The results from the second study partially confirmed those from the first one, showing an overall positivity rate of 3.8% and farm-level prevalence of 19.7% (13). In Thailand, PCV4-DNA positive samples were found in only 1 of 18 evaluated provinces, with low positivity rates of 0.4% at the sample level and 2.07% at the farm level (19). The positivity rate reported from Malaysia was also low at 4.08% (20).

The recently published papers on the detection of PCV4 genetic material outside Asia indicate a broader global distribution of this novel PCV (9, 10, 14). In Spain, PCV4-positive samples were detected exclusively in pigs raised under semi-extensive farming systems, with 7% of digestive samples (intestines, faeces and rectal swabs) and 3.5% of lung samples testing positive (9). In contrast, available data indicate the absence of PCV4 genetic material in samples collected from pigs raised on intensive farms (9). In a study conducted in the United States, 8.6% of porcine samples sent for routine diagnostic investigation from different states tested positive in qPCR (14).

Apart from domestic pigs, PCV4 circulation has also been confirmed in wild boars, with positive samples detected in China and Spain (9, 10, 11, 27). In a study evaluating the presence of PCV4 DNA in Chinese wild boars, 19.6% of the analysed tissue samples tested positive (27). Moreover, three studies revealed a high positivity rate among samples obtained from wild boars in mid-western Spain (9, 10, 11). In the first study, PCV4 DNA was detected in 34.3% of tested wild boars (9). In the remaining two studies, 56 out of 166 and 62 out of 302 evaluated lymph nodes tested positive, and viral genetic material of PCV4 was detected in samples from wild boars originating from each of 7 provinces from which samples were obtained (10, 11). The results of Holgado-Martín *et al*. (10) indicate a slow but progressive spread of PCV4 within Spain. Furthermore, they suggest a more significant role for this country in the spread of PCV4; however, the scarcity of data and existing knowledge gaps do not permit this to be a firm assumption.

Reaching similar findings to ours, some researchers have also failed to detect PCV4 DNA in samples from pigs and wild boars (serum, lymph nodes, lungs, faeces, tissues from aborted foetuses and stillborn piglets) (1, 4, 5, 24). The lack of PCV4 DNA in samples collected from wild boars in Poland is consistent with the previously published results of Frant *et al*. (4). In that study, mainly serum samples, but also spleens, lungs, kidneys, lymph nodes and tonsils from 680 wild boars which were found dead, road-killed or culled between 2018 and 2021 in 14 of the 16 provinces were subjected to qPCR and yielded negative results (4). Similarly, no evidence of PCV4 DNA has been reported in samples from pigs or wild boars in Italy or Catalonia in Spain (5). A study performed in Colombia did not provide any evidence of PCV4 circulation in pigs raised in the country during 2015–2016 or 2018–2019 (24). The results of Anukool and Yamasakul (1) indicating the absence of PCV4 circulation in pigs from northern regions of Thailand contrasted with a previous finding of the presence of the virus in the same area (19); however, the detected presence was only 0.4%. To date, studies evaluating PCV4 circulation have been conducted in only nine countries. Moreover, no one has yet successfully isolated PCV4 from clinical samples. Therefore, a significant knowledge gap exists regarding the geographic distribution and epidemiology of PCV4, highlighting the importance and necessity of further research in this area.

Genetic material of PCV4 has also been detected in species other than *Suidae* representatives, including cattle (28), cats (29), dogs (32) and several species of fur animals, namely raccoon dogs, foxes and minks (26). Therefore, like PCV2 and PCV3, PCV4 is likely to infect a broader range of animal species (22). We decided to evaluate three species of wild ruminants for the presence of PCV4 genetic material because relevant available data were limited. The choice of these species was made based on two primary considerations. The first one is that in Poland, these species and wild boars share a habitat. Wild boars are reservoirs of various pathogens that can be transmitted to other species (6). The risk of transmission from wild boars is rising because the European wild boar population is growing; for example, their number in Poland has doubled during the last 15 years (16). The growth in wild boar populations increases opportunities for contact with other species, especially those occupying the same habitat. The second reason for choosing the selected three deer species is that other PCVs have been detected in some (2, 3). In the present study, we did not detect PCV4 DNA in samples collected from the evaluated wild ruminant species, suggesting that PCV4 is not circulating in Poland. A limitation of this study, however, is the low number of sampled wild deer. Therefore, further research involving larger sample sizes is required to determine the potential circulation of PCV4 in these species.

The results of the present study suggest that PCV4 is not circulating in domestic pigs or wild boars or ruminants in Poland. This assumption is particularly well supported in the case of wild boars, as our findings are consistent with those published by Frant *et al*. (4). However, as previously highlighted by Franzo *et al*. (5), results can be compromised by sample type and the limitations of laboratory techniques, and low virus prevalence can make probable the failure to detect infected animals in the population. Therefore, the following limitations should be addressed. The exact pathogenesis and tissue tropism of PCV4 remain unknown. Choosing an adequate sample type that accurately represents the viral target can be challenging. Regarding pigs, some studies have shown that the highest PCV4 positivity rate is found in lymph nodes (21, 30). In one study, the highest PCV4 viral loads were observed in lymph nodes and the spleen (30). In a study conducted in China, the lungs had the highest detection rate for viral genetic material among the tested porcine samples (12). Porcine circovirus 4 DNA is often detected in serum, which is among the most commonly used samples in studies. In wild boars, PCV4 was detected in the serum, lymph nodes, spleen, kidney and lung samples, while brain and liver samples were negative for the virus (9, 10, 11, 27). The majority of studies assessing the circulation of PCV4 in wild boars used lymph nodes as samples, among which relatively high positivity rates were observed (9, 10, 11, 27). In one study, however, the highest positivity rate was observed in the kidney (27). All the mentioned sample types were included in the study if available. However, investigating only selected tissue samples is another study limitation, as the precise tropism of PCV4 remains unknown.

Regarding pigs, the number of animals included in the present study should have enabled the detection of PCV4 if the virus circulated in the country, even with low prevalence. As previous studies in Europe have shown, whether absence or presence of PCV4 DNA in pig samples is found may depend on the type of farm sampled. In our study, only intensive-breeding farms were included. In a Spanish study aimed at detecting PCV4 circulation in pigs, samples from both intensive and semi-extensive farms were included, and PCV4 genetic material was only detected in pigs from the latter (9). Holgado-Martín *et al*. (10) suggest that the lack of PCV4 DNA detection in samples from the European intensive farm which was sampled may be due to a combination of host, environmental and management factors, as well as pathogen competition. It has been confirmed that PCV4 can infect pigs across different age groups. Viral genetic material and antibodies have been detected in samples from piglets, weaners, fatteners and sows (7, 12). Therefore, samples from all age groups were included in the study.

As already mentioned, one limitation of our study is the small number of wild animals included and the fact that samples from these species were collected only from one region, which may not reflect the epidemiological situation across the country. However, results regarding wild boars support a previous study from Poland (4) and contribute to a more comprehensive understanding of PCV4 circulation in Polish wild boars.

## Conclusion

Although this study found no PCV4 DNA in samples from Poland, further research using a broader range of samples from different animal species – including domestic pigs and wildlife – is needed to monitor the epidemiological situation in the country, especially in light of emerging confirmations of the virus’ presence in Europe.
